# Analysis of Aetiological Agents in Infectious Endocarditis in the Central Military Emergency University Hospital “Dr. Carol Davila” Bucharest

**DOI:** 10.3390/microorganisms12050910

**Published:** 2024-04-30

**Authors:** Corina-Ioana Anton, Ion Ștefan, Simona Mihaela Dumitrache, Alexia-Teodora Ștefan, Diana Răduț, Claudiu-Eduard Nistor, Aurelian-Emil Ranetti, Carmen Adella-Sîrbu, Florentina Ioniță-Radu

**Affiliations:** 1 Department of Infectious Diseases, ‘Dr. Carol Davila’ Central Military Emergency University Hospital, 134 Calea Plevnei, 010242 Bucharest, Romania; corina-ioana.anton@drd.umfcd.ro (C.-I.A.); diana.radut@yahoo.com (D.R.); 2 Department of Medico-Surgical and Prophylactic Disciplines, Titu Maiorescu University, 040441 Bucharest, Romania; 3 Faculty of General Medicine, Carol Davila University of Medicine and Pharmacy, 020021 Bucharest, Romania; teodora.stefan0720@stud.umfed.ro (A.-T.Ș.); ncd58@yahoo.com (C.-E.N.); aurelian.ranetti@umfcd.ro (A.-E.R.);; 4 Thoracic Surgery Department, Carol Davila University of Medicine and Pharmacy, 020021 Bucharest, Romania; 5 Endocrinologic Department, Carol Davila University of Medicine and Pharmacy, 020021 Bucharest, Romania; 6 Clinical Neurosciences Department, Carol Davila University of Medicine and Pharmacy, 050474 Bucharest, Romania; 7 Academy of Romanian Scientists, 50041 Bucharest, Romania; 8 Department of Gastroenterology, ‘Dr. Carol Davila’ Central Military Emergency University Hospital, 134 Calea Plevnei, 010242 Bucharest, Romania

**Keywords:** infective endocarditis, etiology, prosthetic heart valve, multidisciplinary approach, secondary diagnosis

## Abstract

Background: Infective endocarditis (IE) is a pathological condition caused by various microbial agents that can lead to severe complications affecting the heart. Accurate diagnosis is crucial for the effective management of patients with IE. Blood culture is the gold standard for identifying the primary infectious agents, which is a key factor in diagnosing IE using the modified Duke criteria. Objective: The main objective of this study was to investigate the distribution of the etiological agents of IE and the most common secondary diagnoses associated with it. Method: A total of 152 patients aged 23–95 years with a diagnosis of IE and proven etiology (through blood cultures or serological tests) were included in this study. Results: The most common etiological agent identified through blood tests was *Enterococcus faecalis*, which was detected in 39 patients (23.5%). *Staphylococcus aureus* was the second most common agent and was identified in 33 patients (19.9%), followed by Staphylococcus epidermidis, which was identified in 12 patients (13.1%). Nine patients (5.8%) had high levels of anti-*Coxiella burnetti* IgG phase I and II antibodies. Conclusions: IE is a leading cause of death in the Department of Infectious Diseases. Early and accurate diagnosis, along with interdisciplinary treatment, can significantly increase the chances of patient survival. Currently, *Enterococcus faecalis* and *Staphylococcus aureus* are the dominant etiological agents of IE, highlighting the need to revise protocols for prophylaxis, diagnosis, and initial treatment of this condition.

## 1. Introduction

Infective endocarditis (IE) is a rare condition that is difficult to diagnose and often requires associated medical and surgical treatments. It presents an increased incidence of mortality despite the important medical and surgical advances made in the last decade [1,2].

An important contribution to this idea was described in the literature in the last decade, which shows that half of the hospitalized patients die of IE complications [3,4].

Early identification of the infectious etiological agent of IE leads to the optimization of anti-infective treatment in patients. Thus, blood culture remains the gold standard in the diagnosis of infective endocarditis [5,6,7].

In rare cases, when difficult-to-cultivate etiological agents are involved, data on etiology can be obtained using serological tests and gene amplification for infectious agents such as *Coxiella burnetti* or *Bartonella henselae*.

The anti-infective treatment is well-codified in relation to the etiology of IE and the type of valve on which it develops (natural or prosthetic heart valve). In rare situations, it is adjusted according to the impossibility of using a certain antibiotic (for patients who have allergic reactions, or pathologies associated with renal insufficiency). The benefit–risk ratio of surgical interventions is the result of a treatment plan that includes a cardiologist, an infectious disease specialist, and a cardiovascular surgeon [5,6,7].

A multidisciplinary approach for success in optimizing the treatment is frequently performed for patients who present to the Central Military Emergency University Hospital “Dr. Carol Davila”, Bucharest. This type of IE management aims to confirm the diagnosis and develop an optimized diagnostic and therapeutic approach [8,9,10,11].

Early diagnosis, as well as the establishment of a correct interdisciplinary treatment, increases the chances of a favorable evolution for almost all categories of patients with IE [12,13,14].

We performed a retrospective cohort study to evaluate the distribution of etiological agents in IE and the main secondary diagnosis associated with IE among patients admitted to the Central Military Emergency University Hospital “Dr. Carol Davila”, Bucharest.

## 2. Material and Method

This research aimed at carrying out a descriptive analysis of 152 patients with IE who were hospitalized at the Central Military Emergency University Hospital “Dr. Carol Davila”, Bucharest, between 1 January 2017–31 December 2022 and were between 23 and 95 years old.

The inclusion criteria were as follows:

- Patients with a proven etiology (through blood cultures and/or serological tests); and
- Patients with transesophageal ultrasound findings for vegetations;

Patients who did not have a cardiac image evaluation suggestive of vegetation and those who had an implanted cardiac medical device other than those used for valve repair were excluded.

The study initially included 355 patients, of whom 152 met the inclusion criteria and were ultimately included in the analysis.

After obtaining consent from the patients, the data collected included demographic information, patient history, echocardiographic analysis, echocardiographic parameters, drug therapy, and the presence of implantable cardiac devices.

The diagnosis of IE was consistent with the algorithm of the European Society of Cardiology, considering the modified Duke criteria, including those for image evaluation.

For all patients included in the present study, two or three sets of specimens were collected for blood culture testing in special vials: one vial for aerobic bacteria and one for anaerobic bacteria, both of which were inoculated simultaneously in each set. The specimens were collected from venous blood. The punctures were performed at different locations. The collections were performed at minimum intervals of 30 min within a maximum of 24 h from the first collection.

This study was approved by the ethics committee of the Central Military Emergency University Hospital “Dr. Carol Davila”, Bucharest—562/20.12.2022.

## 3. Data Management and Statistical Analysis

Frequency tables and descriptive statistics (mean and standard deviation) were used to analyze patient-related information.

To highlight the differences according to sex and age among the medical characteristics of the patients, two non-parametric statistical tests were applied: the Mann–Whitney test and the Kruskal–Wallis test. These non-parametric tests have the primary advantage of not making assumptions about the shape of the distribution of the population from which the samples were drawn.

The results of the Kruskal–Wallis test were expressed using the H-statistic and the *p*-value. The *p*-value represents the probability of obtaining differences as large as or larger than those observed in our data if the null hypothesis is true. If the *p*-value was less than the predefined significance level of 0.05, we rejected the null hypothesis and concluded that there were significant differences between at least two groups.

The statistical analysis was performed using IMB SPSS Statistics Version 26.

## 4. Results

A total of 152 patients were enrolled in this study. The distribution of patients per year was as follows: 22 patients in 2017 and 2022, 26 in 2018, 29 in 2019, 28 in 2020, and 31 in 2021.

In terms of age, the majority of the patients fell within the range of 61–80 years old, accounting for 63% of the total. Of the patients, 23.8% were between the ages of 41 and 60 years old, while only 6% were over 80 years old. The remaining 7.2% of patients were under the age of 40 years old, with the youngest patient being 23 years old and the oldest being 95 years old, with an average age of 65 years old.

## 5. Analysis of the Distribution of Aetiological Agents

In 143 patients, the etiological agents of IE that were isolated from their blood cultures were identified. In nine of these patients, *Coxiella burnetti* was identified in serological examinations through the detection of IgG phase I and phase II antibodies. No further studies were carried out on the endocardium where surgical intervention was required.

The distribution of infectious agents was as follows:

In 23.5% of the cases, the main etiologic agent was *Enterococcus faecalis*, followed by *Staphylococcus aureus* (19.9%).

For the nine patients included in the present study, phase I and phase II IgG antibodies against *Coxiella burnetii* were detected in the serum.

The analysis of the distribution of infectious agents in 143 patients with positive blood cultures revealed that the most common agents were (Table 1)

- Enterococcus faecalis;
- Staphylococcus aureus;
- Staphylococcus epidermidis;
- Streptococcus sanguinis;
- Streptococcus mitis; and
- Streptococcus gordonii.

The analysis of infectious agents according to the sex of the patients revealed a similar prevalence of the most important infectious agents among both men and women, with *Enterococcus faecalis* being more prevalent among men and *Staphylococcus aureus* more prevalent among women (Table 1).

**Table 1:** Distribution of infectious agents according to patient sex.

		Gender	
		Male	Female
Results	*Enterobacter cloacae*	0.0%	2.0%
	*Enterococcus avium*	1.1%	4.1%
	*Enterococcus faecalis*	**28.7%**	**24.5%**
	*Enterococcus* spp.	0.0%	2.0%
	*Escherichia coli*	1.1%	12.2%
	*Klebsiella pneumoniae*	1.1%	0.0%
	*Pseudomonas aeruginosa*	1.1%	0.0%
	*Coagulase-negative Staphylococci (CoNS)*	1.1%	0.0%
	*Staphylococcus aureus*	**20.2%**	**28.6%**
	*Staphylococcus epidermidis*	8.5%	8.2%
	*Staphylococcus hominis*	1.1%	0.0%
	*Streptococcus agalactiae*	0.0%	2.0%
	*Streptococcus constellatus*	1.1%	0.0%
	*Streptococcus gallolyticus*	4.3%	4.1%
	*Streptococcus gordonii*	9.6%	2.0%
	*Streptococcus mitis*	**11.7%**	0.0%
	*Streptococcus pseudoporcinus*	1.1%	0.0%
	*Streptococcus salivarius*	1.1%	0.0%
	*Streptococcus sanguinis*	7.4%	**10.2%**

The fact that the occurrence of infectious agents is similar between both sexes implies that the likelihood of contracting these agents is comparable between the two sexes, which is significant for addressing and managing infections in the medical context.

The higher occurrence of *E. faecalis* among men may highlight particular risk factors or biological traits that may incease the susceptibility of male patients to this infection.

This could be useful in identifying preventive measures or appropriate treatment plans. In this study, the risk factors that predisposed males to *E. faecalis* infection were age (73% were >60 years old) and the existence of a prosthetic valve (65% of male patients with *E. faecalis* had a prosthetic valve). The higher prevalence of *Staphylococcus aureus* in women suggests that there are female-specific exposure factors. It is crucial to further investigate these findings to understand why this agent appears to affect women more than men.

The analysis of infectious agents based on the age category (Table 2) revealed that the most prevalent infectious agents in each age category, in terms of percentage distribution, were as follows:

For the age category “21–40 years old:”

- *Staphylococcus aureus*, with a prevalence of 33.3%; and
- *Streptococcus sanguinis,* with a prevalence of 22.2%.

For the age category “41–60 years old:”

- *Staphylococcus aureus,* with a prevalence of 20.7%.
- *Enterococcus faecalis* and *Staphylococcus epidermis*, with a prevalence of 17.2%.

For the age category “61–80 years old:”

- *Enterococcus faecalis*, with a prevalence of 34.7%; and
- *Staphylococcus aureus,* with a prevalence of 21.1%.

For the age category “81+ years old:”

- *Staphylococcus aureus,* with a prevalence of 40.0%; and
- *Streptococcus mitis,* with a prevalence of 20.0%.

This provides a comprehensive overview of the infectious agents that are typically identified in various age groups. It is crucial to emphasize that this analysis represents a percentage distribution and does not establish a direct causal relationship between the factors; it only highlights the associations observed in the data.

**Table 2:** Distribution of infectious agents according to patient age.

	AGE				
	≤20	21–40	41–60	61–80	81+
*Enterobacter cloacae*	0.0%	0.0%	0.0%	1.1%	0.0%
*Enterococcus avium*	0.0%	0.0%	3.4%	2.1%	0.0%
*Enterococcus faecalis*	0.0%	0.0%	**17.2%**	**34.7%**	10.0%
*Enterococcus* spp.	0.0%	0.0%	0.0%	1.1%	0.0%
*Escherichia coli*	0.0%	0.0%	3.4%	5.3%	10.0%
*Klebsiella pneumoniae*	0.0%	0.0%	0.0%	1.1%	0.0%
*Pseudomonas aeruginosa*	0.0%	11.1%	0.0%	0.0%	0.0%
*Coagulase-negative Staphylococci (CoNS)*	0.0%	0.0%	0.0%	1.1%	0.0%
*Staphylococcus aureus*	0.0%	**33.3%**	**20.7%**	**21.1%**	**40.0%**
*Staphylococcus epidermidis*	0.0%	0.0%	**17.2%**	7.4%	0.0%
*Staphylococcus hominis*	0.0%	11.1%	0.0%	0.0%	0.0%
*Streptococcus agalactiae*	0.0%	0.0%	0.0%	1.1%	0.0%
*Streptococcus constellatus*	0.0%	11.1%	0.0%	0.0%	0.0%
*Streptococcus gallolyticus*	0.0%	11.1%	0.0%	5.3%	0.0%
*Streptococcus gordonii*	0.0%	0.0%	13.8%	5.3%	10.0%
*Streptococcus mitis*	0.0%	0.0%	10.3%	6.3%	**20.0%**
*Streptococcus pseudoporcinus*	0.0%	0.0%	3.4%	0.0%	0.0%
*Streptococcus salivarius*	0.0%	0.0%	0.0%	1.1%	0.0%
*Streptococcus sanguinis*	0.0%	**22.2%**	10.3%	6.3%	10.0%

## 6. Analysis of Secondary Diagnoses According to Age, Agent, and Presence or Absence of Valvular Prosthesis

**Figure 1:**
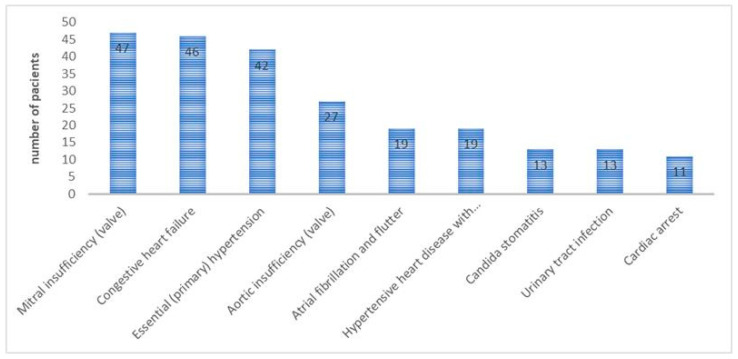
The distribution of the most prevalent secondary diagnoses among patients who test positive for blood cultures.

Among patients with blood cultures (n = 143), most registered the following secondary diagnoses (Figure 1):

- Mitral valves insufficiency 32.8%;
- Congestive heart failure 32.1%;
- Essential (primary) hypertension 29.3%; and
- Aortic valve insufficiency 5.2%.

Depending on the age category, the distribution of secondary diagnoses highlights that, in the cases of the first 10 most common secondary diagnoses, the most affected age group was 61–80 years old, followed by the 41–60 age group (Table 3).

The analysis of the distribution of the three most prevalent secondary diagnoses, namely, mitral valve insufficiency, congestive heart failure, and essential (primary) hypertension, were associated with the age group 61–80 years old in a proportion of approximately 65%.

In the case of the diagnosis of hypertensive cardiomyopathy with congestive heart failure, there was an increase in the prevalence of this diagnosis among people aged between 41–60 years old (42.1%) and among people aged between 61 and 80 years old (52.6%). Among the patients with a secondary diagnosis of cardiac arrest, 84.6% belonged to the age group 61–80 years old.

Approximately 7% of patients with associated diagnoses, such as candidal stomatitis, fall within the age group of 21–40 years old. Notably, this specific diagnosis was considered in the context of antibiotic therapy.

The distribution of the type of agent in the analysis of the results of the 143 patients highlights that the most common infectious agents were:

- Enterococcus faecalis;
- Staphylococcus aureus;
- Staphylococcus epidermidis;
- Streptococcus sanguinis;
- Streptococcus mitis; and
- Streptococcus gordonii.

The analysis of secondary diagnoses according to the type of agent (Table 4) revealed that the most prevalent agents associated with the vast majority of secondary diagnoses were *Enterococcus faecalis* and *Staphylococcus aureus*.

In the secondary diagnoses of mitral (valve) insufficiency, aortic (valve) insufficiency, and hypertensive heart disease with (congestive) heart failure, the first two infectious agents identified were *Staphylococcus aureus* and *Enterococcus faecalis.* In the case of essential hypertension, atrial fibrillation and flutter, urinary tract infection or cardiac arrest, the highest incidence was recorded for *Enterococcus faecalis* followed by *Staphylococcus aureus*.

Patients with aortic insufficiency and cardiac arrest had high incidences of the infectious agent *Streptococcus gordonii*, while those with hypertensive heart disease with congestive heart failure and atrial fibrillation and flutter had *Streptococcus sanguinis* as the causative agent (Table 4).

The most prevalent secondary diagnoses in patients with native valves were mitral valve insufficiency, congestive heart failure, and essential (primary) hypertension (Table 5).

## 7. Characteristics of Patients with Positive Blood Cultures

These statistics provide data for 143 patients and their distribution according to two different characteristics: age category and sex of the patient.

**Age category:**

- 9 patients (6.3%) were between 21 and 40 years of age;
- 29 patients (20.3%) were between 41 and 60 years of age;
- 95 patients (66.4%) were between 61 and 80 years of age; and
- 10 patients (7.0%) were 81 years of age or older.

**Patient gender:**

- male: 94 patients (65.7%)
- female: 49 patients (34.3%)

These data provide an overview of the distribution of patients according to age and gender. It can be seen that the majority of patients in this group (66.4%) are between 61 and 80 years of age and that more men (65.7%) than women (34.3%) were included in the study.

## 8. Analysis of Secondary Diagnoses According to Age and Infectious Agent

Among the patients with anti-*Coxiella burnetti* IgG phase I and phase II antibodies (n = 9), most had secondary diagnoses of mitral (valve) regurgitation and congestive heart failure.

Table 6 provides an overview of the distribution of patients according to age groups and based on two variables: age and two additional diagnoses, namely, mitral valve insufficiency and congestive heart failure. This distribution is presented as a percentage of the total number of patients in each age group.

For the age groups 41–60 and 61–80 years old, both had secondary diagnoses of mitral valve insufficiency and congestive heart failure, which exhibited uniform distributions across both of these two age categories. This finding indicates that the two secondary diagnoses were equally prevalent in these age groups.

It is worth noting that no cases were registered for either of the two secondary diagnoses in the age categories of “≤20” and “81+”. In other words, no instances of mitral valve insufficiency or congestive heart failure were observed in these two age groups.

## 9. Analysis of the Characteristics of Patients with anti-*Coxiella burnetti* IgG Phase I and Phase II Antibodies

This study presents the dispersion of individuals with anti-*Coxiella burnetti* IgG phase I and phase II antibodies, along with suggestive values for the diagnosis of IE. The study was conducted using two variables: age and sex of the patients included in the analysis. The results are presented in terms of the absolute number of patients and the percentage of patients within each category.

Age:

- Most patients (55.6%) fall into the 61–80 age group;
- Two patients (22.2%) were identified for the age groups 21–40 and 41–60 years old; and
- No patients over 80 years of age with anti-*Coxiella burnetii* IgG phase I and II antibodies were identified.

Regarding the sex of the patients included in this study, it was found that, of the total number of patients with phase I and phase II anti-*Coxiella burnetti* IgG antibodies present in the serum, 77.8% were male and 22.2% were female.

It can be seen that this diagnosis is more common between the ages of 41 and 80 years old, more predominantly in the male patients than in the female patients.

## 10. Results

Thise article presents an analysis of blood cultures from a retrospective cohort study of 143 patients and a retrospective cohort study of nine patients in whom phase I and phase II *Coxiella brunetti* IgG antibodies were detected in the serum, all of whom were diagnosed with infective endocarditis and admitted to the military emergency university hospital “Dr. Carol Davila”, Bucharest.

This study identified the infectious etiological agents by performing blood cultures for 143 patients, and nine of the hospitalized patients developed anti-*Coxiella burnetii* IgG phase I and phase II antibody titers.

Two or three sets of special blood culture vials were collected from all of the patients; they were collected separately for both the aerobic and anaerobic infectious agents and both cultures were inoculated simultaneously. The punctures were performed from venous blood from different locations each time, and the collections were made at minimum intervals of 30 min for a maximum of 24 h.

It is important to note that, within the Central Military Emergency University Hospital “Dr. Carol Davila”, Bucharest, most blood cultures revealed *Enterococcus faecalis* bacteria as the main infectious agent; 39 patients among those included in this study had two or three positive blood cultures with this bacterium (23.5%), followed by positive blood cultures with the bacteria *Staphylococcus aureus* (33 patients—19.9%), and *Staphylococcus epidermidis* and *Streptococcus sanguinis* in equal percentages (13.1%), correlating with the data we found in the literature [15,16].

Among patients with positive blood cultures, the most common diagnoses were mitral valve insufficiency, with a prevalence of 32.8%; congestive heart failure, with a prevalence of 32.1%; essential (primary) hypertension, with a prevalence of 29.3%; and aortic valve insufficiency, with a prevalence of 18.8%, while among patients with anti-*Coxiella Brunetti* IgG phase I and phase II antibodies present in the serum, the secondary diagnoses with the highest prevalence were mitral valve insufficiency and insufficiency in congestive heart rate in equal percentages.

The collected data coincided with those in the literature, suggesting a predisposition for cardiac damage in the evolution of infective endocarditis.

Depending on the age category, the distribution of the secondary diagnoses highlights the fact that, in the case of patients with positive blood cultures, secondary diagnoses with the highest prevalence affect patients in the 61–80 years of age group, followed by the 41–60 years of age group

The analysis of the distribution of the three most prevalent secondary diagnoses, namely, mitral valve insufficiency, congestive heart failure, and essential (primary) hypertension, were associated with the age group of 61–80 years old in a proportion of approximately 63%. In the case of hypertensive cardiomyopathy with congestive heart failure, there was an increase in the prevalence of this diagnosis among people aged between 41–60 years old (42.1%) and among people aged between 61 and 80 years old (52.6%). Most patients with a secondary diagnosis of cardiac arrest (84.6%) were within the age group of 61–80 years old.

Approximately 7% of the patients with associated diagnoses, such as candidal stomatitis, were within the age group of 21–40 years old. The analysis of secondary diagnoses according to the type of agent revealed that the most prevalent agents associated with the vast majority of secondary diagnoses were *Enterococcus faecalis* and *Staphylococcus aureus.*

In the secondary diagnoses of mitral valve insufficiency, aortic valve insufficiency, and hypertensive heart disease with congestive heart failure, the first two infectious agents identified were *Staphylococcus aureus* and *Enterococcus faecali;* in the case of essential hypertension, atrial fibrillation and flutter, urinary tract infection, or cardiac arrest, the highest incidence was recorded for *Enterococcus faecalis,* followed by *Staphylococcus aureus*.

In the case of candidal stomatitis, the most common agents were *Streptococcus sanguinis* and *Streptococcus mitis*. Patients with aortic insufficiency and cardiac arrest had high incidences of the infectious agent *Streptococcus gordonii*, whereas those with hypertensive heart disease, congestive heart failure, and atrial fibrillation, and flutter were most frequently associated with *Streptococcus sanguinis*.

For patients with phase I and phase II anti-*Coxiella brunetti* IgG antibodies, the distribution of secondary diagnoses by age category showed that mitral valve insufficiency and congestive heart failure were evenly distributed in the 41–60 and 61–80 years of age groups. This suggests that these two secondary diagnoses are equally prevalent in these age groups, a conclusion that is also reflected in the literature.

The Kruskal–Wallis test results, which investigate variations in the prevalence of secondary diagnoses across the primary age categories, indicate significant differences in cases of mitral valve insufficiency and hypertensive heart disease with congestive heart failure among the different age groups. Specifically, the probability associated with the test is below the maximum significance threshold of 10%. Consequently, patients aged between 41 and 60 are the most likely to have secondary diagnoses such as mitral insufficiency and hypertensive heart disease.

The Mann–Whitney U Test, also known as the Wilcoxon Rank–Sum Test, was utilized to evaluate whether there were any discernible differences between the groups of patients infected with specific pathogens. For *Enterococcus faecalis*, the *p*-value of 0.918 is greater than the conventional significance level of 0.05 (or 5%). Conversely, for *Staphylococcus aureus*, the *p*-value of 0.047 is below the standard significance level, indicating that there are significant differences between the patient groups with infective endocarditis caused by this pathogen.

For *Streptococcus gordonii*, the *p*-value of 0.023 is less than the conventional significance level, suggesting that there are significant differences in the incidence of this pathogen among patients. In contrast, the *p*-values for *Staphylococcus epidermidis*, *Staphylococcus sanguinis*, *Staphylococcus mitis*, and *Staphylococcus gordonii* are greater than 0.05, indicating that there are no significant differences in their incidence among patient groups.

In summary, the results of the Mann–Whitney U Test indicate that there are significant differences in the incidences of *Staphylococcus aureus* and *Streptococcus gordonii*, while there are no significant differences for *Enterococcus faecalis* or the other pathogens examined.

The analysis of the distribution of infectious agents resulting from blood cultures according to age category highlighted that the most prevalent infectious agents for the age category of 21–40 years old were *Staphylococcus aureus,* with a prevalence of 33.3%, and *Streptococcus sanguinis,* with a prevalence of 22.2%. For individuals aged 41–60 years old, the most common bacteria was *Staphylococcus aureus*, with a prevalence of 20.7%, and *Enterococcus faecalis* and *Staphylococcus epidermidis*, with a prevalence of 17.2%. For those aged 61–80 years old, the most frequent agents were *Enterococcus faecalis*, with a prevalence of 34.7%, and *Staphylococcus aureus*, with a prevalence of 21.1%. In the age group of 81 years old and above, the main culprits were *Staphylococcus aureus* (40.0% prevalence) and *Streptococcus mitis* (20.0% prevalence).

The analysis of the infectious agents resulting from the blood cultures according to the sex of the patient revealed that 94 patients (65.7%) were male, and 49 patients (34.3%) were female.

When analyzing of the secondary diagnoses according to the sex of the respondent, in the patients in whom the presence of anti-*Coxiella burnetti* IgG phase I and phase II antibodies were determined, it was found that for the patients with a secondary diagnosis of mitral valve insufficiency, all patients were male.

In this study, the sex distribution of patients with anti-*Coxiella burnetti* IgG phase I and II antibodies were examined. It was observed that, out of the total number of patients, 77.8% were male, while 22.2% were female. These findings indicate that this diagnosis is more prevalent among patients aged 41–80 years old and is more common in male patients than in female patients. These results should be considered when formulating multidisciplinary therapeutic protocols for infective endocarditis. The main purpose of this research was to analyze the distribution of etiological agents in IE at the Military Emergency University Hospital “Dr. Carol Davila”, Bucharest and to examine the characteristics of the patients included in the study.

This analysis aims to provide a comprehensive grasp of the incidence and features of this condition within the aforementioned medical institution. This study employs a sample comprising 152 patients diagnosed with infective endocarditis from the Military Emergency University Hospital “Dr. Carol Davila”, Bucharest. Considering the shifting etiology of infective endocarditis over the past decade, it has become imperative to reassess the prophylaxis and diagnostic procedures for IE.

## 11. Discussion

The most relevant epidemiological characteristics of the group were as follows: the incidence was similar each year, the average age of the patients was 61 years old, and the male-to-female ratio was 3:1. These results were similar to those reported in other studies [17].

In the present study, the etiology and risk factors of IE caused by various incriminating agents were analyzed. Positive hemocultures with infectious agents such as *S. aureus* and *E. faecalis* were correlated with IE and patient outcome [15].

During the 5-year study that we conducted, *E. faecalis* was the most frequently detected incriminating agent. This finding is not correlated with the findings of other authors that suggest that *S. aureus* was the most frequently detected incriminating agent [17].

In this study, *S. aureus* was the second leading cause of IE, a very aggressive pathogen which is often associated with a very high mortality rate [7,14,17,18].

When the data on secondary diagnoses were summarized, a significant number of patients had IE associated with mitral valve insufficiency and congestive heart failure. Echocardiography was performed for diagnostic purposes. There have been previous studies in which patients were diagnosed with IE-associated secondary heart problems [8,18].

Among the nine patients included in this study who presented with anti-*Coxiella burnetti* phase I and phase II antibodies in the serum, we noted that all of them developed IE and 88.9% had prosthetic heart valves and associated secondary heart problems. Chronic infection occurs almost exclusively in patients with pre-existing valvular heart disease and prosthetic heart valves [16].

Our observations indicate that the majority of patients were male (77.8%), which aligns with the findings of other studies [12,13,14].

## 12. Limitations

The present study had some limitations. This study was performed in a single hospital, and multicenter studies with more patients may reveal information regarding multiple etiologies. Long-term monitoring of patients is also important for a better assessment of prognosis. The lack of serological determinations was also a limitation of the present study.

Nine patients had negative blood cultures but developed anti-*Coxiella burnetti* phase I and phase II antibodies on serological tests.

We propose that patients with infective endocarditis benefit from more accurate clinical evaluation and early diagnosis in at-risk populations. Serial laboratory serological tests and transthoracic and transesophageal echocardiography should be performed in patients with suspected endocarditis after clinical evaluation.

## Figures and Tables

**Table 3:** Distribution of most common secondary diagnoses according to age.

AGE (Binned)					
≤20		21–40	41–60	61–80	81+
**Mitral valve** **insufficiency**	0.0%	6.4%	31.9%	57.4%	4.3%
**Congestive heart failure**	0.0%	2.2%	28.3%	63.0%	6.5%
**Essential (primary) hypertension**	0.0%	0.0%	16.7%	73.8%	9.5%
**Aortic valve insufficiency**	0.0%	0.0%	15.8%	68.4%	15.8%
**Atrial fibrillation and flutter**	0.0%	3.7%	14.8%	77.8%	3.7%
**Hypertensive heart disease with (congestive) heart failure**	0.0%	0.0%	42.1%	52.6%	5.3%
**Candidal stomatitis**	0.0%	7.7%	23.1%	53.8%	15.4%
**Urinary tract infection**	0.0%	0.0%	27.3%	63.6%	9.1%
**Heart attack**	0.0%	0.0%	15.4%	84.6%	0.0%

**Table 4:** Distribution of secondary diagnoses accordingto the infectious agent type.

	*Enterococcus* *faecalis*	*Staphylococcuss* *aureus*	*Staphylococcuss* *epidermidis*	*Streptococcus* *sanguinis*	*Streptococcus* *mitis*	*Streptococcus* *gordonii*
**Mitral valve insufficiency**	17.0%	23.4%	8.5%	12.8%	10.6%	8.5%
**Congestive heart failure**	21.7%	15.2%	10.9%	10.9%	10.9%	8.7%
**Essential(primary) hypertension**	35.7%	21.4%	4.8%	7.1%	4.8%	7.1%
**Aortic (valve) insufficiency**	15.8%	21.1%	5.3%	5.3%	10.5%	15.8%
**Atrial fibrillation and flutter**	29.6%	22.2%	14.8%	11.1%	7.4%	3.7%
**Hypertensive heart disease** **with (congestive) heart failure**	15.8%	21.1%	15.8%	15.8%	15.8%	10.5%
**Candida stomatitis**	15.4%	15.4%	7.7%	23.1%	23.1%	7.7%
**Urinary tract infection**	27.3%	18.2%	9.1%	9.1%	27.3%	9.1%
**Heart attack**	30.8%	23.1%	0.0%	0.0%	0.0%	15.4%

**Table 5:** The prevalence of secondary diagnoses in individuals with native valves.

Secondary Diagnoses	%
**Mitral (valve) insufficiency**	36.9%
**Congestive heart failure**	32.3%
**Essential (primary) hypertension**	24.6%
Aortic (valve) insufficiency	13.8%
Atrial fibrillation and flutter	20.0%
Hypertensive heart disease with (congestive) heart failure	12.3%
Candida stomatitis	9.2%
Urinary tract infection	4.6%
Cardiac arrest	9.2%

**Table 6:** Distribution of the main secondary diagnoses according to age category.

	AGE				
	≤20	21–40	41–60	61–80	81+
Mitral valve insufficiency	0.0%	0.0%	50.0%	50.0%	0.0%
Congestive heart failure	0.0%	0.0%	50.0%	50.0%	0.0%

## Data Availability

The data presented in this study are available upon request from the corresponding author. The data are not publicly available due to the confidentiality of health data.
